# Decoding Cosmetic Complexities: A Comprehensive Guide to Matrix Composition and Pretreatment Technology

**DOI:** 10.3390/molecules29020411

**Published:** 2024-01-15

**Authors:** Xiao-Nan Du, Yu He, You-Wen Chen, Qian Liu, Lei Sun, Hui-Min Sun, Xian-Fu Wu, Yong Lu

**Affiliations:** National Institutes for Food and Drug Control, Beijing 102629, China; xnd@live.com (X.-N.D.); heyu9942@126.com (Y.H.); chenyouwen_bucm123@163.com (Y.-W.C.); 3321010492@stu.cpu.edu.cn (Q.L.); dasunlei@sina.com (L.S.); sunhm@126.com (H.-M.S.)

**Keywords:** cosmetics, personal care products, compositions, pretreatment, detection, regulation

## Abstract

Despite advancements in analytical technologies, the complex nature of cosmetic matrices, coupled with the presence of diverse and trace unauthorized additives, hinders the application of these technologies in cosmetics analysis. This not only impedes effective regulation of cosmetics but also leads to the continual infiltration of illegal products into the market, posing serious health risks to consumers. The establishment of cosmetic regulations is often based on extensive scientific experiments, resulting in a certain degree of latency. Therefore, timely advancement in laboratory research is crucial to ensure the timely update and adaptability of regulations. A comprehensive understanding of the composition of cosmetic matrices and their pretreatment technologies is vital for enhancing the efficiency and accuracy of cosmetic detection. Drawing upon the China National Medical Products Administration’s 2021 Cosmetic Classification Rules and Classification Catalogue, we streamline the wide array of cosmetics into four principal categories based on the following compositions: emulsified, liquid, powdered, and wax-based cosmetics. In this review, the characteristics, compositional elements, and physicochemical properties inherent to each category, as well as an extensive overview of the evolution of pretreatment methods for different categories, will be explored. Our objective is to provide a clear and comprehensive guide, equipping researchers with profound insights into the core compositions and pretreatment methods of cosmetics, which will in turn advance cosmetic analysis and improve detection and regulatory approaches in the industry.

## 1. Introduction

With the development of the economy and people’s pursuit of beauty, the cosmetics industry has experienced significant growth [1,2]. Despite stringent regulations, such as China’s Cosmetic Safety Technical Specifications and the European Union’s Cosmetic Regulation 1223/2009, which prohibit hazardous ingredients—including lead, mercury, arsenic, antibiotics, hormones, and carcinogens [3,4,5]—illicit products continue to infiltrate the market, posing potential dermatological risks and severe health hazards to consumers [6,7,8,9,10]. According to the China National Medical Products Administration, 601 batches of non-compliant cosmetics were detected in 2023, 79 of which contained unauthorized ingredients [11]. These products with illegal additives span various categories, including acne treatment cosmetics that were found with hormones like dexamethasone and clobetasol propionate and antibacterial drugs, such as clindamycin, lincomycin, ofloxacin, ciprofloxacin, chloramphenicol, and metronidazole. Similarly, hair care products contained preservatives like methylchloroisothiazolinone and methylisothiazolinone, along with hair dye agents such as p-aminophenol and 2-chloro-phenylenediamine sulfate. Furthermore, face masks have been found to include hormones like desonide and flumethasone and nail polishes were found with prohibited solvents such as dichloromethane and 1,2-dichloroethane. In addition, skin lightening products have been found to contain hormone-like clobetasol propionate, and shampoos have been found to include the preservative triclosan. Even baby creams have been found to contain the illegal antifungal agent terbinafine. These illegal additives, while cost-effectively enhancing product claims, pose significant health and environmental risks.

The reasons for this phenomenon are multifaceted. Primarily, commercial interests undeniably motivate manufacturers. Additionally, the intrinsic complexity of cosmetics poses significant challenges to detection, allowing dishonest merchants to exploit vulnerabilities. Cosmetic matrices, comprising a complex blend of preservatives, emulsifiers, and thickeners, necessitate effective pretreatment before analytical detection, especially given the diverse and minute quantities of illicit additives [12,13,14,15]. Although the selection of pretreatment techniques is not strictly constrained by the type of sample matrix, each matrix type has its most suitable pretreatment method. Consequently, an insufficient understanding of the compositions and properties of the matrices may lead to inappropriate pretreatment and inadequate enrichment of illegal additives. For instance, solid-phase extraction (SPE) has been employed in the pretreatment of various cosmetic products. However, SPE was initially developed as a complement or replacement for liquid–liquid extraction (LLE), making it more suited for the analysis of liquid cosmetics [16,17]. In the case of non-liquid cosmetics, it is imperative to consider the specific matrix composition of the sample and incorporate additional processing steps by which to circumvent issues such as column clogging, extended processing times, excessive solvent use, and low recovery rates [18]. Similarly, the matrix solid-phase dispersion (MSPD) method involves mixing the sample with dispersing and solid-phase extraction agents, followed by physical grinding to ensure thorough contact between the analytes and the solid-phase extractor. This method is primarily applicable to solid and semi-solid samples, rather than liquid cosmetics. Inappropriate selection of pretreatment methods can lead to reduced recovery rates, potentially resulting in illegal additives being omitted during detection and regulatory evasion. Therefore, a comprehensive understanding of the composition and characteristics of different cosmetic matrices is crucial when selecting appropriate pretreatment techniques and achieving effective regulation.

**Figure 1:**
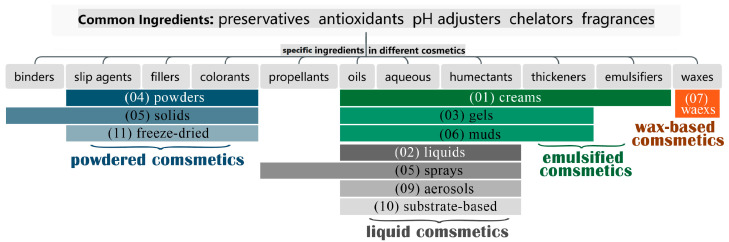
Classification and compositions of cosmetic dosage forms.

The current literature summarizes cosmetic pretreatment methods based primarily on the type of extraction method, meticulously detailing the evolution and optimization of various treatment techniques [17,18,19]. However, these articles often overlook a significant issue: the considerable differences in the matrix components of different cosmetic formulations, which hinders researchers from effectively selecting suitable pretreatment methods without a thorough understanding of the composition and properties of the cosmetic matrix. A rigorous scientific approach in this field should focus on the compatibility of both extraction methods and cosmetic ingredients. However, the multifaceted nature of cosmetics analysis, encompassing cosmetic science, pharmaceutical chemistry, and analytical chemistry, presents technical and informational gaps as hurdles, leading to a lack of comprehensive literature reviews that address matrix compositions and pretreatment techniques tailored to each cosmetic category. The Cosmetic Classification Rules and Classification Catalogue, introduced by the National Medical Products Administration of China in 2021 categorizes cosmetics into 12 types based on the following formulations: creams, liquids, gels, powders, solids, muds, waxes, sprays, aerosols, substrate-based cosmetics, freeze-dried cosmetics, and others (Figure 1) [20]. Our review condenses these into four primary categories based on composition and examines their matrix ingredients, key compounds, and physicochemical characteristics. We also summarize the advances in pretreatment methods for different categories that have been developed since 2005. The objective is to provide researchers with a detailed understanding of the various cosmetic matrices and aid in the refinement of pretreatment research. Such advancement is essential for enhancing the detection and monitoring of illegal additives, reinforcing the security of cosmetic products.

The structure of the rest of this paper is as follows: Section 2 presents the compositions and representative compounds of cosmetics, in which we discuss the common and specific ingredients across different categories of cosmetics, along with their representative compounds. This section also delves into the mechanisms of action, physicochemical properties, additive thresholds, and pretreatment methods of these compounds.

Section 3 presents advances in cosmetic pretreatment techniques, in which we delineate the evolution of pretreatment strategies for each cosmetic category since 2005. In the conclusion and the Future Perspectives Section, we synthesize the main content of this article and provide a prospective introduction to emerging pretreatment technologies, aiming to offer guidance and perspectives for future research.

## 2. Compositions and Representative Compounds of Cosmetics

In the latest “classification rules”, many cosmetic forms share similar ingredients. Sprays and aerosols, for instance, are distinguished mainly by the presence of propellants, yet their base formulations are akin to liquid cosmetics [21]. Creams and gels characteristically contain significant quantities of humectants and thickeners, while powders, solids, and freeze-dried cosmetics predominantly feature powdery solids [22]. Considering that cosmetics with similar ingredients can use comparable pretreatment processes, this review proposes a categorization that simplifies 12 dosage forms into the following four primary categories: emulsified, liquid, powdered, and wax-based cosmetics, as depicted in Figure 1. This reclassification facilitates a clearer comprehension of the commonalities and distinctions within cosmetic matrices, thus fostering the innovation of targeted and efficient pretreatment techniques in cosmetic science.

### 2.1. Common Ingredients in Cosmetic Formulations

Despite the marked variations in the matrices of diverse cosmetics, the universal application of preservatives, antioxidants, pH adjusters, chelators, and fragrances pervades various cosmetic categories [22].

#### 2.1.1. Preservatives

**Figure 2:**
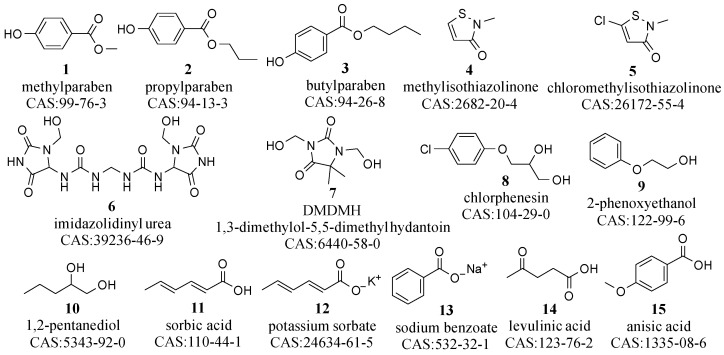
Key preservatives utilized in cosmetic formulations.

Pursuant to European Regulation 1223/2009, a preservative is defined as “any substance aimed at inhibiting the proliferation of microorganisms in cosmetics”, thus extending the product’s shelf life [18]. Common preservatives include parabens (compounds **1**–**3** in Figure 2), isothiazolinones (compounds **4**–**5**), formaldehyde releasers (compounds **6**–**7**), ether alcohols (compounds **8**–**10**), and organic acids alongside their salts (compounds **11**–**15**), as shown in Figure 2 [23]. Parabens, such as methyl-, propyl-, and butylparaben, are cost-effective and widely used for their antifungal and antibacterial properties, especially in rinse-off products [24]. Isothiazolinones, like methylisothiazolinone, offer broad-spectrum antibacterial activity and remain effective across diverse pH levels [25]. Formaldehyde releasers, including imidazolidinyl urea and dimethyloldimethyl hydantoin (DMDMH), ensure low levels of free formaldehyde, providing microbial protection [26]. Ether alcohols, such as chlorphenesin, 2-phenoxyethanol, benzyl alcohol, pentanediol, hexanediol, and octanediol, recognized as gentler preservative alternatives, also offer broad-spectrum antibacterial benefits [27,28,29]. Organic acids and their salts, containing levulinic acid, anisic acid, sorbic acid/potassium sorbate, and benzoic acid/sodium benzoate, although less effective against bacteria, inhibit fungi and are often used in combination with other substances like hydantoin for enhanced preservation [30,31].

Parabens (compounds **1**–**3**) feature a planar benzene ring structure, which facilitates interactions with microbial cell membranes. The ester groups (methyl, ethyl, or propyl) exhibit increased lipophilicity with longer carbon chain lengths, enhancing their ability to penetrate cell membranes and improving their preservative efficacy. The hydroxyl groups primarily enhance water solubility, aiding in the dispersion of these compounds in cosmetic products and diffusion within microbial cells. Additionally, isothiazolinones (compounds **4**–**5**), containing a five-membered isothiazolone ring with sulfur and nitrogen, can react with intracellular biomolecules such as proteins and enzymes, thereby disrupting the normal functions of microbial cells. Moreover, ether alcohol (compounds **8**–**10**), typically containing an ether group (-O-) and one or more hydroxyl groups (-OH), exhibit a balance of hydrophilicity and lipophilicity. This unique structure facilitates the penetration of microbial cell membranes and disrupts cellular metabolic processes, which contributes to their bactericidal action. These compounds can also interact with the lipids in cell membranes, leading to membrane disruption and leakage of cellular contents. Finally, the preservative activity of organic acids and their salts (compounds **11**–**15**) is primarily achieved by altering the pH within microbial cells, thereby disrupting their metabolic processes. Undissociated organic acids penetrate microbial cell membranes and dissociate within the cell due to the higher internal pH, releasing protons and acidifying the internal environment. The salt forms of organic acids, typically more water-soluble than the acids themselves, are more suitable as preservatives in aqueous formulations.

The structures of these compounds not only impart unique functionalities but also determine their individual solubility characteristics. Parabens, ethers, and isothiazolinones are predominantly lipid- and organic-solvent soluble, whereas formaldehyde releasers, alcohols, and organic acids demonstrate enhanced water solubility. This variance underscores the importance of suitable pretreatment techniques in different cosmetic detection.

#### 2.1.2. Antioxidants

**Figure 3:**
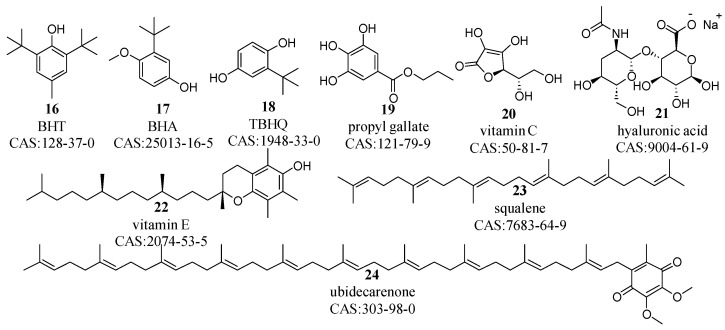
Principal antioxidants in cosmetic formulations.

The complexity of cosmetic formulations, with their oxidization-prone components, such as lipids, requires an antioxidant system for quality preservation and product stability [32]. Antioxidants, as shown in Figure 3, are broadly categorized into synthetic and natural types [33,34,35]. Synthetic antioxidants (compounds **16**–**19**), such as butylated hydroxytoluene (BHT), butylated hydroxyanisole (BHA), tertiary-butylhydroquinone (TBHQ), and propyl gallate, are engineered in the laboratory to effectively inhibit oxidative damage from light, heat, or metals. In contrast, natural antioxidants (compounds **20**–**24**)—sourced mainly from plants, fruits, or other biological origins—include vitamin E, vitamin C, superoxide dismutase (SOD), coenzyme Q10, and other compounds, such as curcumin, astaxanthin, resveratrol, glutathione, rosemary extracts, and various flavonoids.

Among these antioxidants, compounds **16**–**22**, which contain phenolic hydroxyl groups, are capable of capturing lipid free radicals, thereby disrupting lipid peroxidation processes [36]. These groups impart water solubility to these compounds. However, their solubility in organic solvents, such as ethanol, xylene, and chloroform, can be enhanced by increasing the number of hydroxyl groups or modifying the substituents on the phenyl ring. Compounds **23** and **24**, characterized by their long-chain polyconjugated systems, are effective at neutralizing singlet oxygen and free radicals, consequently reducing oxidative stress [37]. Additionally, their long carbon chain structures confer increased lipophilicity. Notably, vitamin C’s high antioxidant capacity is tempered by its instability and potential skin irritancy, prompting the use of its derivatives in cosmetic products.

#### 2.1.3. pH Adjusters

**Figure 4:**
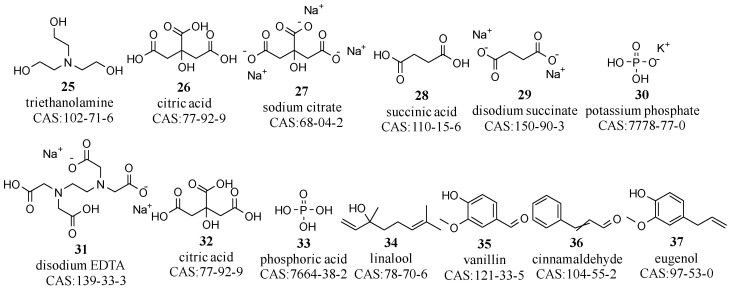
Predominant pH adjusters, chelators, and fragrances in cosmetic matrices.

Most cosmetic products have a pH range between 4.0 and 8.5. The distinct acid–base properties of certain ingredients necessitate the use of pH adjusters to maintain the pH within this desired range [38,39]. Common pH adjusters include sodium hydroxide (NaOH), potassium hydroxide (KOH), triethanolamine, citric acid, sodium citrate, succinic acid, disodium succinate, and potassium phosphates (compounds **25**–**30** in Figure 4) [40,41]. Certain components, such as the high-molecular-weight thickener carbopol, require specific pH conditions to function optimally. In this case, an alkaline pH adjuster is needed for effective thickening. With advances in material technology, there is now a diverse selection of over a hundred thickening agents available. pH adjusters are thus essential for the stability, safety, and performance of cosmetic products.

NaOH and KOH, as strong alkaline substances, effectively neutralize acidic components and are commonly used in products requiring higher pH levels, such as soaps and facial cleansers. In contrast, triethanolamine, a weaker base, is primarily used to reduce the pH of products to more closely match the skin’s natural pH, making it more suitable for use in skin care products. Additionally, citric acid, sodium citrate, succinic acid, and disodium succinate are often combined as buffering agents to regulate and maintain the pH in formulations with specific pH requirements. NaOH, KOH, and sodium citrate dissociate into ions in water, while triethanolamine and citric acid can form hydrogen bonds with water molecules. Consequently, these pH adjusters exhibit good water solubility.

#### 2.1.4. Chelators

Chelators are crucial in cosmetics for binding metal ions, preventing deleterious interactions with other ingredients, and enhancing product stability and longevity [42,43]. Disodium ethylenediaminetetraacetic acid (EDTA) is a common chelator that not only effectively immobilizes various metal ions but also exhibits a synergistic interplay with preservatives and antioxidants. Although allergic responses are undocumented, disodium EDTA may facilitate skin penetration because of its low molecular weight, which could disrupt the metal ion balance in the body and cause irritation or other negative effects [44]. Other notable chelators include citric acid and phosphoric acid (compounds **31**–**33** in Figure 4).

Chelators possess chemical structures capable of forming multiple coordination bonds with metal ions. For example, disodium EDTA, with its four carboxyl and two amino groups, establishes six-point coordination, forming stable cyclic structures with metal ions. Similarly, citric acid forms multipoint coordination via its three carboxyl and one hydroxyl groups. These functional groups significantly contribute to their water solubility.

#### 2.1.5. Fragrances

Cosmetic matrices incorporate fragrances to impart a desirable scent. These fragrances are broadly classified into natural and synthetic categories [45]. Natural fragrances, sourced from botanical and zoological materials, such as petals, leaves, roots, fruits, resins, and animal-derived essences, endow products with authentic scents. In contrast, synthetic fragrances, engineered in laboratories, have complex compositions formed by combining various chemical structures [46]. The Catalogue of Cosmetic Ingredients in Use (2021 Edition) lists only four substances explicitly as fragrances: flavor (06156), aroma (07007), parfum (07008), and fragrance (08782). It also lists compounds, including linalool, vanillin, cinnamaldehyde, and eugenol, that can be used as fragrances (compounds **34**–**37** in Figure 4) [47].

Cosmetic products encompass not only common ingredients, such as preservatives, antioxidants, pH adjusters, chelators, and fragrances, but also specific ingredients that impart distinctive properties and effects to each product. In pretreatment research, it is imperative to consider these ingredients to ensure the precision and efficiency of the research process. This review proceeds to delineate four cosmetic formulation categories, detailing their respective compositional nuances.

### 2.2. Emulsified Cosmetics

Emulsified cosmetic categories—encompassing creams, gels, and muds—are the primary vehicles for various skincare formulations such as face creams, facial cleansers, and aloe vera gels. These matrices are intricate, consisting of oils, aqueous phases, emulsifiers, humectants, and thickeners, in addition to their common ingredients [48,49]. The synergistic interplay of these ingredients determines the specific properties and performance efficacy of emulsified cosmetics.

#### 2.2.1. Oils

**Figure 5:**
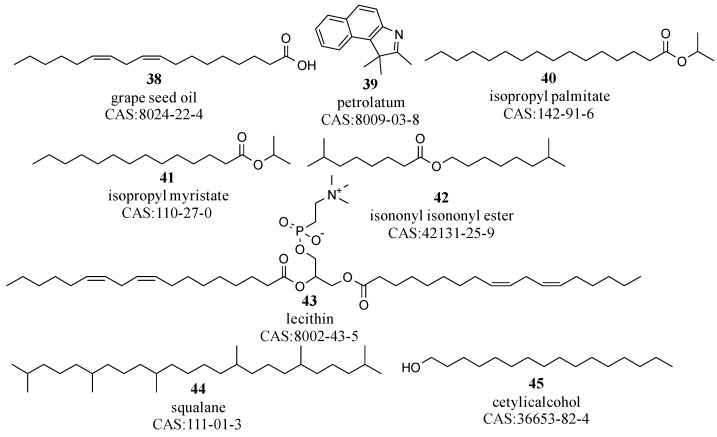
Key oil-based constituents in cosmetic formulations.

Oil-based ingredients confer softness, lubricity, and spreadability to emulsified cosmetics, primarily consisting of plant oils such as shea, olive, and grape seed oil (compound **38** in Figure 5); animal fats such as lanolin and beeswax; mineral fats such as petrolatum (compound **39**); synthetic fats, including isopropyl palmitate and caprylic/capric triglyceride (compounds **40**–**42**); silicones, notably, polydimethylsiloxane; and fatty alcohols such as lecithin, squalane, and cetylicalcohol (compounds **43**–**45**), as shown in Figure 5 [50,51,52,53].

Oil-based ingredients feature long carbon chains, which enhance their lipophilicity, allowing for effective integration with other non-polar substances. Additionally, the hydrophobic nature of the long carbon chains in these oil-based ingredients aids in forming a protective layer on the skin surface, inhibiting moisture evaporation, and thereby serving a crucial lubricating function in cosmetic products [54,55]. These ingredients are soluble in nonpolar solvents such as alkanes meaning that n-hexane or petroleum ether are suitable solvents for their extraction during pretreatment.

#### 2.2.2. Aqueous Phases

Deionized water is the most common aqueous phase in cosmetics, primarily dissolving water-soluble preservatives, antioxidants, and active agents such as botanical extracts, fermentation broths, and enzymatic formulations [56]. The International Cosmetic Ingredient Standard Chinese Name Catalog (2010) mainly lists thermal spring water and seawater among its entries. However, niche waters, including deep sea [57] and glacial varieties [58], have recently entered the market, and are desired for their rich mineral contents and potential soothing, anti-inflammatory, and antioxidative properties.

#### 2.2.3. Emulsifiers

**Figure 6:**
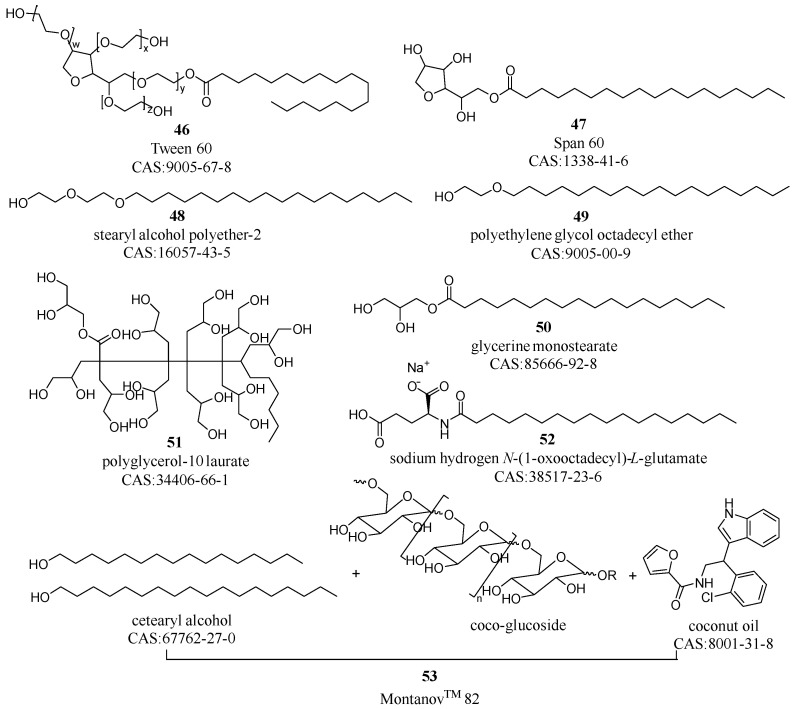
Central emulsifying agents in cosmetic preparations.

Emulsifiers are crucial for stabilizing emulsified cosmetics, with their selection depending on factors such as their oil-to-water ratios, hydrophilic–lipophilic balance (HLB) values, and critical packing parameters [59,60]. Prominent emulsifiers include Tween and Span, specifically, Tween 60 for oil-in-water (O/W) emulsions and Span 60 for water-in-oil (W/O) emulsions (compounds **46**–**47** in Figure 6); fatty alcohol polyoxyethylene ethers such as stearyl alcohol polyether-2 and polyethylene glycol octadecyl ether (compounds **48**–**49**); glycerides such as polyoxyethylene stearate and glycerine monostearate (compound **50**); polyglycerines such as polyglyceryl-10 derivatives (compound **51**); anionic types such as sodium aliphatic alcohol sulfate (compound **52**); and saccharide derivatives, exemplified by Montanov^TM^ 202 and 82 (compound **53**), as shown in Figure 6 [61,62,63].

Emulsifiers are characterized by their amphiphilic nature, with the hydrophilic portion typically comprising polar functional groups such as hydroxyl groups (compounds **46–51**), carboxyl groups, or charged groups (compound **52**). Conversely, the hydrophobic part usually consists of non-polar structures, such as long carbon chains (compounds **46–50**) or hydrophobic cyclic structures (compound **53**). The amphiphilic nature of emulsifiers contributes to their emulsion stability but also presents challenges in cosmetic pretreatment [64]. Their complex structures and high molecular weights make gel chromatography a suitable method for their separation and removal, which is particularly effective for analytes with lower molecular weights [65,66].

#### 2.2.4. Humectants

**Figure 7:**
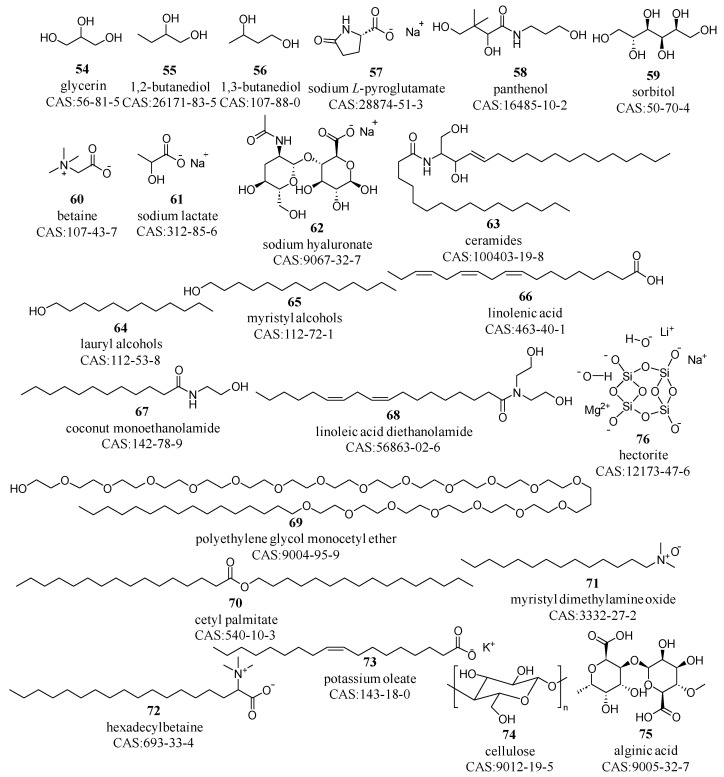
Prominent humectants and thickeners employed in cosmetic matrices.

Humectants, such as polyols like glycerin, butylene glycol, and panthenol; natural skin components, such as sodium hyaluronate, trehalose and ceramides; and amino acid derivatives, including betaine, sodium pyrrolidone carboxylic acid and sodium lactate [67,68] (compounds **54**–**63** in Figure 7), are essential for retaining moisture, preventing desiccation, and maintaining the skin’s hydration equilibrium [69].

Humectants contain multiple hydroxyl, carboxyl, or other polar groups, which can form hydrogen bonds with water molecules, thereby absorbing and retaining moisture. Low molecular weight humectants (compounds **54**–**61**) can penetrate the skin, providing hydration to deeper layers. In contrast, high molecular weight humectants, such as sodium hyaluronate (compound **62**), form a hydrating film on the skin surface, effectively locking in moisture and reducing water loss. Humectants can form hydrogen bonds with water molecules and also engage in intermolecular hydrogen bonding, endowing them with unique water solubility, high melting and boiling points, and significant viscosity [70]. During the pretreatment process, liquid–liquid extraction methods can be employed to separate these humectants from the liquid cosmetics.

#### 2.2.5. Thickeners

The texture of emulsified cosmetic formulations is largely influenced by thickeners, which are second only to solvents in terms of quantity [71]. Thickeners are categorized by their functional groups: inorganic salts such as sodium chloride and sodium phosphate; fatty alcohols and acids, including lauryl and myristyl alcohols, as well as linolenic acid (compounds **64**–**66**); alkanolamides, such as coconut monoethanolamide and linoleic acid diethanolamide (compounds **67**–**68**); ethers, such as polyethylene glycol monocetyl ether (compound **69**); esters, such as isostearate and cetyl palmitate (compound **70**); oxidized amines, such as myristyl dimethylamine oxide (compound **71**); amphoteric surfactants, including cocamidopropyl and hexadecyl betaine (compound **72**); anionic surfactants, such as potassium oleate (compound **73**); cellulose derivatives, such as cellulose and its gums (compound **74**); natural gums and modified gums, such as alginates and guar gum (compound **75**); and inorganic polymers and their derivatives, for instance, magnesium aluminum silicate, montmorillonite, and hectorite (compound **76**), as shown in Figure 7 [72,73,74,75].

Most thickeners are composed of long carbon chains, along with polar groups such as hydroxyl (compounds **64**, **65**, **69**), carboxyl (compounds **66**, **75**), and amide groups (compounds **67**, **68**). These long-chain structures intertwine within solutions, increasing the solution’s resistance to flow and thereby enhancing its viscosity. Additionally, the polar groups can form hydrogen bonds with water molecules, further enhancing the water’s viscosity. Thickeners can be categorized into two main types based on their solubility: water-soluble and oil-soluble [76,77]. Water-soluble thickeners are primarily composed of polar groups, whereas oil-soluble thickeners contain long carbon chains. In the pretreatment research of cosmetics, the selection of an appropriate solvent depends on the specific type of thickener used.

### 2.3. Liquid Cosmetics

In this context, liquid cosmetics refer to products without emulsifiers and exhibit significant fluidity, including liquids, sprays, aerosols, and substrate-based cosmetics, such as shampoos, perfumes, nail polishes, and facial masks. The compositions of these products are relatively uncomplicated, primarily comprising common ingredients, solvents, and humectants, as well as propellants, which are characteristic of spray products.

#### 2.3.1. Solvents

Solvents are essential in the formulation of liquid cosmetics, as they dissolve active ingredients and enhance product fluidity. Commonly used solvents include water, ethanol, ethyl acetate, toluene, oils, and botanical extracts [78,79,80,81,82]. Water is often preferred due to its low cost and high solvency. Ethanol is effective in dissolving oils, fragrances, and plant-derived ingredients and is frequently used with water to leverage its antimicrobial properties. Ethyl acetate and toluene are staples in nail polish, though concerns about toluene’s irritation potential have led to its decreased use and regulatory restrictions internationally [83]. Oils and plant extracts are selected not only for their solvency but also for their additional benefits to the product.

#### 2.3.2. Propellants

Propellants are essential for the ejection of contents from aerosol containers, such as hairspray. Common propellants include propane, butane, isobutane, and environmentally friendly options such as carbon dioxide and nitrogen [84,85]. The use of hydrofluorocarbons (HFCs) and hydrochlorofluorocarbons (HCFCs) is severely restricted because of their ozone-depleting effects [86,87]. Consequently, the choice of propellants is dictated not only by the specific needs of the product but also by stringent regional environmental regulations.

### 2.4. Powdered Cosmetics

Powdered cosmetics encompass powders, solids, and freeze-dried cosmetics such as body powders, pressed powders, and blushes. These products are formulated with binders, slip agents, fillers, and colorants, in addition to their common ingredients, which collectively determine the texture, appearance, and performance of the cosmetic item [88].

#### 2.4.1. Binders

In powdered cosmetics, binders are essential for combining slip agents, fillers, colorants, and other materials into a cohesive product [89]. Natural binders, such as beeswax and keratin, not only help to hold the product together but also provide antimicrobial benefits. Oleophilic binders, such as liquid paraffin and silicone oils, are chosen for their ability to enhance the smoothness of application and to stabilize the particles. Synthetic binders, such as polymethyl methacrylate (PMMA), are favored for their strong cohesive properties and for imparting a flexible and sleek texture to the final product [90,91].

#### 2.4.2. Slip Agents

In powdered cosmetic formulations, slip agents, such as sorbitan esters, propylene glycol, and PEG-8, play a crucial role in improving texture and facilitating smooth application. These ingredients help to optimize powder dispersion, prevent the excessive cohesion of particles, and reduce the likelihood of caking, thereby promoting an even application and imparting a silky feel to the skin [92]. Due to their hygroscopic properties, which allow them to attract and retain moisture, they also contribute to maintaining skin hydration [93].

#### 2.4.3. Fillers

In powdered cosmetics, fillers, such as mica, kaolin, calcium carbonate, talc, silica, pearl, and nylon, are used to modulate color intensity, increase volume, and enhance texture. These substances confer beneficial characteristics, such as sheen for visual appeal, silkiness for a smooth touch, oil absorption for longer wear, and soft-focus effects for a flattering finish [94,95]. However, their chemical stability and low solubility can pose challenges during pretreatment processing, as these properties may necessitate specialized techniques for their dispersion and dissolution.

#### 2.4.4. Colorants

Colorants are pivotal in powdered cosmetics for providing hue and are categorized as organic or inorganic. Organic colorants encompass plant-based pigments, synthetic dyes, and lakes, which are dyes deposited onto substrates. In contrast, inorganic colorants, such as iron oxides, zinc oxide, titanium dioxide, and mica, are often preferred in such formulations for their stability, minimal skin irritation, and ability to provide opacity and coverage [96,97]. These inorganic options are favored not only for their consistent coloration but also for their safety profile and non-irritating nature, ensuring both product efficacy and consumer comfort [98].

### 2.5. Wax-Based Cosmetics

Wax-based cosmetics are products that incorporate the waxes, such as carnauba, candelilla, jojoba, bees, and Japan waxes, that are essential for enhancing stability, controlling viscosity, and ensuring a non-tacky feel [99]. Carnauba wax is particularly prized in color cosmetics for its excellent thickening and stabilizing abilities. Candelilla wax is treasured for its natural composition, contributing to product consistency and a refined texture. Jojoba wax is recognized for its remarkable stability and resistance to oxidation, making it a versatile choice for a variety of wax-based products. Beeswax is widely used for its inherent stability and antimicrobial properties, making it a staple in color cosmetics as well as skincare preparations [100,101,102,103]. The pronounced hydrophobic properties of wax signify its insolubility in water. However, wax is only soluble in certain organic solvents, such as chloroform, mineral oil, or certain alcohols. Additionally, the inherent viscosity of wax further complicates its pretreatment process.

### 2.6. Thresholds for Cosmetic Ingredient Additions

Cosmetics, as complex mixtures composed of various ingredients, benefit from an understanding of their approximate compositional ranges to optimize pretreatment and analytical conditions. For instance, in the application of preservatives, chlorphenesin (compound **8**) is permitted at a maximum concentration of 0.3%, whereas 1,2-pentanediol (compound **10**) can reach up to 21.29% in the highest historical usage for residency category products. Awareness of such information is crucial when identifying and mitigating potential interferences during pretreatment and analysis. We conducted a thorough inquiry into the additional ranges of each substance mentioned in the aforementioned section. Acknowledging that different countries and regulations may vary in their requirements for the same substance, our initial references were the European Union regulations (EC) No 1223/2009 [104] and Commission Regulation (EU) 2022/2195 [105], followed by the Safety and Technical Standards for Cosmetics of China (2015 Edition) [106] and the Cosmetics Ingredients Catalog used in China (2021 edition) [107]. Additionally, we consulted data from several professional websites (Table 1).

### 2.7. Illegal Additives in Cosmetics

Beyond the complexity of their matrices, cosmetics also present intricate challenges due to the illegal additives incorporated into them. In China, the “Prohibited Ingredients Catalogue for Cosmetics” and the “Prohibited Plant and Animal Materials Catalogue in Cosmetics” cumulatively prohibit the use of 1393 ingredients [125], while the European Union’s EC No. 1223/2009 regulation lists 1328 banned substances [104]. However, the actual number of illicitly added ingredients far exceeds those enumerated in these directories. The reasons for this are multifaceted. Firstly, all compounds with similar functions are uniformly classified under the same category, such as “antibiotics” and “substances with androgenic effect”. Secondly, derivatives and salts of certain compounds are also grouped together, for instance, “aniline, its salts and its halogenated and sulphonated derivatives”. Lastly, these prohibitory lists are continuously updated, not only adding new banned substances but also shifting the status of certain ingredients from prohibited to restricted. These factors collectively amplify the complexity and challenges in the regulation of cosmetics.

The utilization of illegal additives in cosmetics is driven by a myriad of complex factors. Initially, some manufacturers might opt for prohibited, lower-cost chemical substances as substitutes for expensive, yet safe, ingredients. For instance, illegal additives N-(5-Chlorobenzoxazol-2-yl)acetamide and alkyne alcohols are inexpensive and have antimicrobial and antiseptic effects. Additionally, substances like benzidine, antimony, arsenic, and xylidines, despite their pigmentary properties and lower costs, are employed as alternative pigments. Furthermore, certain illegal additives are incorporated into cosmetics to augment product efficacy. Medications originally intended for anxiety and insomnia treatment, such as carbromal, isocarboxazid, benzazepines, and bromisoval, might be illicitly added to products like frankincense, essential oils, and skincare items for their sedative effects. Hypoglycemic drugs like tolbutamide, carbutamide, and sulfonylurea derivatives, due to their ability to reduce sugar absorption and consequently aid in weight loss, are sometimes used in slimming cosmetics. Spironolactone, an anti-androgen medication, can decrease sebum production, aiding in acne control. Anti-inflammatory drugs such as antibiotics, aminocaproic acid, cinchophen, epinephrine, and mofebutazone, can alleviate skin inflammation and swelling. Trichloroacetic acid, used for exfoliating old skin layers and mitigating hyperpigmentation, is commonly found in acne and skin whitening products. While these drugs possess specific therapeutic effects, their use should be strictly regulated in medical contexts and never added to cosmetics without authorization. Nonetheless, some unscrupulous businesses might incorporate these substances into their products to enhance effectiveness, reduce costs, and increase profit margins. Lastly, the presence of certain illegal additives might result from technical flaws in the production process rather than intentional inclusion. For example, heavy metal contaminants, like lead, mercury, cadmium, and residual solvents, such as anthracene oil and benzene. Although unintentional, these instances pose potential health risks to consumers and thus necessitate stringent regulation.

### 2.8. Analytical Technologies in Cosmetics

The diversity of illegal additives determines the crucial role of selecting appropriate analytical technologies. Ultraviolet/visible spectroscopy (UV/VIS) primarily operates on the principle of substance absorption of ultraviolet or visible light, utilizing absorption spectra for the analysis of compounds. It is particularly applicable to organic compounds capable of absorbing UV or visible light, such as dyes and sunscreens in cosmetics [126]. Infrared spectroscopy (IR), conversely, is based on the principle of the molecular absorption of specific frequencies of infrared light, causing changes in vibrational energy levels. This method is suitable for both organic and inorganic compounds containing chemical bonds, frequently used in the analysis of lipids, fragrances, and moisturizers in cosmetics [127]. Mass spectrometry (MS) identifies molecular mass and composition by measuring the trajectory of molecules in electric or magnetic fields, applicable to nearly all compounds and particularly suited for the qualitative and quantitative analyses of complex cosmetic components [128]. Nuclear magnetic resonance (NMR) leverages the absorption and emission of radio waves by atomic nuclei in an external magnetic field, applicable to organic compounds containing active nuclei (such as ^1^H, ^13^C), and is instrumental in analyzing the structure of complex organic compounds in cosmetics [129]. Gas chromatography (GC) employs gas as the mobile phase and separates components through a stationary phase in a chromatography column, ideal for volatile and thermally stable organic compounds like fragrances and solvents in cosmetics [130]. High-performance liquid chromatography (HPLC) uses a liquid as the mobile phase under high pressure to separate components, is extensively applicable to various organic compounds, including high molecular weight and non-volatile substances, and is used for detecting and analyzing moisturizers, preservatives, vitamins, and other components in cosmetics [131]. Atomic absorption spectroscopy (AAS) is based on the absorption of specific wavelengths of light by atomic vapor and is used for the analysis of metal elements and some metal compounds, such as heavy metal impurities (like lead or mercury) in cosmetics [132]. Atomic fluorescence spectroscopy (AFS) relies on the fluorescence generated by atoms transitioning between ground and excited states, is primarily used for trace element analysis (such as mercury, arsenic or selenium) and is applicable to the detection of trace harmful elements in cosmetics [133].

These techniques play a vital role in the analysis of cosmetics, ensuring product quality and safety, and aiding in the development of new products. Given the considerable variation in components across different types of cosmetics, selecting appropriate analytical techniques is crucial for the accurate identification and quantification of these components.

In Section 2, we discuss the common ingredients and the specific components of four different types of cosmetic products, along with their mechanisms of action, addition thresholds, and pretreatment methods. For instance, parabens, widely used as preservatives in cosmetics, owe their microbial growth inhibition properties to their phenolic structure, while their ester-linked carbon chains enhance the ability to penetrate cell membranes. The combined effects of these groups endow these compounds with lipophilicity. Another category of commonly used preservatives, organic acids and their salts, exhibit high water solubility due to their ability to form hydrogen bonds with water or to dissociate into ions in water. Additionally, we provide a brief introduction to common illegal additives and analytical techniques. Selecting appropriate pretreatment techniques based on the type of cosmetic and specific components is crucial for a more efficient removal of the cosmetic matrix, aiding in the separation and enrichment of analytes such as illegal additives and restricted compounds. Thus, the following section will summarize the pretreatment methods applicable to different types of cosmetic products.

## 3. Advances in Cosmetic Pretreatment Techniques

The diversity of cosmetic matrices requires appropriate pretreatment methods. For emulsified cosmetics rich in emulsifiers and thickeners, techniques such as field-assisted extraction, which utilizes oscillation or microwave-assisted heating, and supercritical fluid extraction, often with CO_2_, are preferred [134]. Conversely, liquid cosmetics with simpler matrices benefit from phase separation techniques such as liquid–liquid extraction [135,136]. Aqueous extraction is ideal for products rich in polar compounds, while headspace analysis is best suited for isolating volatile constituents [137]. This review delineates the evolution of pretreatment strategies for diverse cosmetic categories since 2005. The advantages, disadvantages, and applicable matrices of the pretreatment techniques discussed in this article are briefly summarized in Table 2.

### 3.1. Emulsified Cosmetics

Emulsified cosmetics, with their intricate matrices of thickeners and emulsifiers, often present challenges in traditional solvent dissolution, resulting in suboptimal analyte recovery. In 2012, Zhong et al. [134] addressed this by employing ultrasound-assisted matrix solid-phase dispersive liquid extraction (UA–MSPD), which utilizes anhydrous sodium sulfite to disperse hair dyes, facilitating the extraction of analytes into an acidic medium. Concurrently, oil-based substances and thickeners are solubilized in n-hexane, with adsorbents capturing interfering compounds. This study innovatively employs dispersants to effectively disperse viscous hair dyes, thereby significantly simplifying the operational process. The method requires only 9 min to complete the entire procedure and demonstrates a high recovery rate, ranging from 85.7% to 107%.

Solid-phase extraction (SPE), a traditional preparation technique, faces limitations when applied to emulsified cosmetics due to mass transfer resistance, which can lead to longer elution times and higher solvent consumption. In 2014, Zhan et al. [5] introduced an improved approach known as dispersive solid-phase extraction (dSPE). This method is initiated by suspending the cosmetic in acetonitrile. Subsequently, C18 silica is added as an adsorbent, and MgSO_4_ is employed to facilitate the dispersion of the matrix, followed by vigorous shaking and cotton filtration. The use of dispersants in conjunction with cotton filtration effectively eliminates interference from complex sample matrices in analytical results, streamlining the extraction process. This approach demonstrates excellent linearity for concentrations of up to 480 g/kg, with correlation coefficients ranging from 0.982 to 0.999.

Bimatoprost and latanoprost, as first-line pharmacological agents for glaucoma treatment, have been determined to induce hypertrichosis as a side effect. Unscrupulous cosmetic manufacturers exploit this adverse effect by incorporating these drugs into eyelash growth serums. Prolonged exposure to cosmetics containing prostaglandin drugs can potentially lead to local skin or ocular discomfort, allergic reactions, and even impact cardiovascular health. Compared with traditional extraction methods, ultrasound-assisted extraction (UAE) demonstrates superior efficiency, gentleness, and environmental friendliness. It effectively extracts active ingredients from complex matrices while preserving the stability of thermosensitive substances. In 2023, Yong et al. [129] applied UAE technology to process commercially available eyelash serums, followed by separation and purification using silica gel columns. Employing the Cosmetic Risk Substance Screening Platform, they detected suspicious components in two different fractions. These components were further purified using high-performance liquid chromatography and identified as bimatoprost and latanoprost through high-resolution mass spectrometry and nuclear magnetic resonance techniques. This extraction method exhibited an excellent linear range from 0.25–50 ng/mL (R_2_ > 0.9992), with notably low detection (LOD) and quantitation limits (LOQ) of 0.01 and 0.03 mg/kg, respectively.

Fullerene, a nanomaterial composed of carbon atoms, is utilized in the cosmetics industry due to its exceptional antioxidative properties. However, given its status as an emerging nanomaterial, it is imperative to conduct a comprehensive and rigorous assessment of its potential impacts and safety. Liquid–liquid extraction (LLE), a common separation technique, often struggles with emulsified cosmetics due to stable emulsions formed by emulsifiers and surfactants, which resist phase separation [64]. In 2006, Xia et al. [138] improved this method by incorporating acetic acid, which effectively dissolves these challenging components, thereby enhancing the recovery of fullerenes from facial creams and serums. This modified approach successfully overcomes the emulsification challenges that typically impede traditional liquid–liquid extraction methods.

### 3.2. Liquid Cosmetics

Liquid cosmetics, with their relatively simple matrix, are well-suited to a variety of extraction techniques, thanks to the minimal risk of emulsification and column clogging. Applicable techniques include LLE, cloud point extraction (CPE), SPE, and stir-bar sorptive extraction (SBSE) [139].

Liquid–liquid extraction is a favored method in analytical chemistry for its efficiency and simplicity [135]. Certain water-compatible organic solvents can separate into two distinct phases in the presence of inorganic salts. Cai et al. [136] efficiently isolated phthalates from cosmetic waters and perfumes using this method, which is notably straightforward, achieves rapid partition equilibrium, and integrates seamlessly with HPLC analysis. CPE, another efficient technique, employs surfactant micellization for phase separation [140,141]. In 2011, Soruraddin et al. [142] utilized this method in treating shampoo with sulfuric acid and hydrogen peroxide, subsequently warming it to 40 °C and adding 0.5% phenol to induce micelle formation, enabling the spectrophotometric determination of trace selenium (IV) within the micellar phase of Triton X-100. This study employs an economical and environmentally friendly approach, utilizing temperature elevation to the cloud point to form micelles, thereby achieving biphasic separation. This method is straightforward in operation, with a recovery rate ranging from 98% to 102%. Additionally, co-precipitation-assisted cloud point extraction enhances this technique by using co-precipitants for better analyte extraction. In 2013, Xiao et al. [143] combined aluminum hydroxide co-precipitation with sodium dodecyl sulfate-mediated cloud point extraction to effectively isolate and extract five estrogens from toners (17β-estradiol, estrone, ethinyl estradiol, diethyl stilbestrol, and dihydro stilbestrol). Similar to CPE, this study enhances the enrichment and separation of target analytes during cloud point extraction by incorporating coprecipitants to form precipitates with the analytes. Applied to the extraction of estrogens in cosmetics, this method achieves a recovery rate ranging from 77.3% to 104.1%.

SPE is extensively utilized in food safety, pharmaceutical analysis, and biomedicine, using common adsorbents like diatomaceous earth, alumina, silica, and C18 and C8 silicas [144,145]. Recently, innovative adsorbents have been developed, such as nitrated garlic skin [146], corn fibers [147], molecularly imprinted polymers [148], artificial antibodies [149], and magnetic SPE. In 2020, Zhao et al. [150] developed a technique for detecting four steroids (ethinylestradiol, norgestrel, megestrol acetate and medroxyprogesterone acetate) in shampoo, employing a deep-eutectic-solvent-based magnetic colloidal gel (DES–MCG) in magnetic SPE. They synthesized a DES–MCG adsorbent using a choline chloride–urea deep eutectic solvent with magnetic multi-walled carbon nanotubes (MMWCNTs). This novel adsorbent was used to extract analytes from concentrated shampoos, achieving recoveries ranging from 80.1% to 118.8%. This method integrates deep eutectic solvents with MMWCNTs, not only optimizing the sample processing procedure but also enhancing the recovery rate. Moreover, it exhibits superior environmental friendliness, aligning with the requirements of green analytical chemistry.

Parabens, common preservatives in cosmetics, are limited by the EU to a maximum concentration of 0.8% (*w*/*w*) in products. Traditional SPE suffers from low selectivity, often resulting in the co-extraction of various matrix components, adversely affecting the quantitative analysis of analytes. Molecularly imprinted polymers (MIP) are synthetic materials capable of selectively interacting with specific chemical functional groups. Utilizing MIPs that selectively bind parabens enhances the selectivity of SPE. In 2018, Vicario et al. [151] synthesized MIPs using propylparaben as a template. They employed MIP-based solid-phase extraction (MISPE) for pretreatment of commercially available baby wipes, followed by analysis using high-performance liquid chromatography (HPLC). This method, demonstrating an over 86.15% recovery rate, showed greater selectivity, stability, and improved retention capabilities compared to traditional SPE methods.

Acrylamide, a compound capable of forming covalent bonds with macromolecules such as proteins and DNA, is known to induce mutations and has potential carcinogenic effects. The presence of amino acids and sugars in cosmetic ingredients can lead to the formation of acrylamide during processing. Consequently, monitoring and controlling the content of acrylamide in cosmetics is of paramount importance. In 2023, Schettino et al. [13] utilized vortex-assisted reverse-phase dispersive liquid phase microextraction (LPME), employing water as the extraction solvent, to successfully extract and pre-concentrate acrylamide from liquid hand soaps and makeup removers. Subsequent analysis of the extracted acrylamide was conducted using liquid chromatography–tandem mass spectrometry (LC–MS/MS). This method, upon validation, demonstrated excellent analytical performance, including linearity, detection and quantification limits, with a recovery rate of 88–108%. Efficient, simple, and rapid, this method precisely quantifies trace levels of acrylamide, providing a robust tool for assessing the safety of cosmetics.

### 3.3. Powdered Cosmetics

Powdered cosmetics, rich in minerals that are largely insoluble in conventional solvents, present challenges in pretreatment. In 2010, Cha et al. [152] tackled this by using a mixture of nitric and hydrofluoric acids for mineral decomposition via microwave digestion, which enabled the quantitative analysis of six metals—iron, copper, zinc, lead, nickel, and cadmium—in various cosmetic products using flame atomic absorption spectroscopy (FAAS). Microwave digestion involves heating a sample with a strong acid inside a closed vessel using microwave radiation. This process significantly speeds up the digestion process, allowing for the efficient breakdown of the sample matrix. Expanding on this technique, Bocca et al. [132], in 2013, employed a similar acid digestion method followed by LC–MS for the measurement of trace metals in face powders. The acid nitration method effectively decomposes both organic and inorganic components in cosmetics, disrupting the complex matrix and insoluble inorganic constituents, thereby facilitating the detection of metal elements and other targeted analytes.

Liquid-phase microextraction (LPME) is a sample preparation technique used to isolate and concentrate analytes from liquid samples. It involves a small amount of an organic solvent, which is immiscible with the aqueous sample, to extract the analytes. In 2007, Xiao et al. [153] developed the LPME–HPLC method for the precise quantification of trace levels of estratriol, estradiol, ethinylestradiol, and estriol in toners. This methodology involved the optimization of various experimental conditions, including the selection and volume of the acceptor phase solvent, stirring speed, and extraction duration. The analysis of these four estrogens using this method yielded recovery rates ranging from 101.2% to 114.9%. Compared with traditional pretreatment methods, this novel approach employed LPME technology, resulting in a substantial reduction in organic solvent usage and a decrease in operation time, thereby offering advantages to simplicity, rapidity, sensitivity, and environmental friendliness.

Matrix solid-phase dispersion (MSPD) offers an efficient alternative to traditional solvent-based sample preparation by blending and grinding the sample directly with an adsorbent, making it particularly effective for solid and semi-solid matrices [17,154,155]. In 2019, Chen et al. [156] developed a protocol utilizing MSPD to detect colorants in solid cosmetics such as blush and eyeshadow. The method involved grinding the cosmetics with anhydrous sodium sulfate and sand, then transferring the mixture to an SPE column, eluting with methanol, and analyzing the eluates using ultra-high-performance liquid chromatography (UHPLC) coupled with quadrupole-orbitrap high-resolution mass spectrometry (Q-orbitrap HRMS). This approach successfully identified 11 prohibited colorants in cosmetics, including acid blue, acid red, acid black, acid orange, acid yellow, solvent green, solvent orange, solvent yellow, pigment red, basic violet and disperse yellow. The study demonstrated the effectiveness of combining desiccants and dispersants (anhydrous sodium sulfate and sand) in sample grinding, significantly enhancing the homogeneity of the sample and the extraction efficiency of the analytes (ranging from 64.0% to 128.4%).

Similar to MSPD, pressurized liquid extraction (PLE) [157] and accelerated solvent extraction (ASE) [158] are commonly used for extracting analytes from powdered cosmetics. The use of dispersants in these methods helps prevent sample compaction and optimizes solvent consumption. This not only enhances the efficiency of the extraction process but also minimizes the environmental footprint.

### 3.4. Wax-Based Cosmetics

The complex waxes in wax-based cosmetics hinder dissolution and separation, necessitating the use of potent yet toxic organic solvents like chloroform or dichloromethane and extensive pretreatment to isolate analytes [98,159].

To overcome the challenges associated with wax-based cosmetics, Wang et al. [160] introduced a method to isolate rhodamine B without using harsh solvents. Their technique utilized mechanical stirring along with sodium lauryl sulfate, an anionic surfactant, to disperse lipstick in water at 333 K (59.85 °C). The mixture was then subjected to SPE using a cotton-packed column to separate waxes, followed by fluorescence detection for quantification. This method effectively removed wax residues by reversing the flow of the mobile phase. This study adopts a straightforward approach, dissolving lipstick in a solvent through mechanical stirring at high temperatures, effectively circumventing complex sample pretreatment steps, thereby significantly enhancing the processing efficiency.

Ashing is a sample preparation process used in analytical chemistry to remove organic components from a sample. In this process, a sample is heated to high temperatures in the presence of air or oxygen, leading to combustion or thermal decomposition of organic substances. The result is a residue primarily composed of inorganic ash, which can then be analyzed for its elemental composition. This method is ideal for isolating heat-stable compounds in wax-based cosmetics. In 2012, Brandão et al. [161] used this technique to determine the lead content in various cosmetics, such as mascara, concealer, lipstick, and lip gloss. Their procedure involved incinerating the cosmetic samples at 600 °C, followed by digestion with nitric acid and quantification using flame atomic absorption spectroscopy for accurate lead measurements.

**Table 2:** The advantages, disadvantages, and applicable matrices of the pretreatment techniques.

Pretreatment Techniques		Advantages	Disadvantages	Applicable Matrices	Refs.
Solid phase extraction	Solid-phase extraction (SPE)	Offers high selectivity, removes complex matrices, and concentrates analytes	Can require multiple processing steps and meticulous optimization of conditions	Water-based or low viscosity cosmetic products	[16]
	Magnetic SPE	Easy to operate, fast, and amenable to automation	Higher cost of magnetic materials	Products with substantial particulates or for rapid sample processing	[150]
	Mechanical stirring SPE	Efficiently mixes the sample, enhancing extraction efficiency	Equipment may be more complex	Samples that require thorough mixing to improve extraction efficiency	[160]
	Selective adsorbent SPE	Targeted extraction of specific components using specific adsorbents	Requires precise selection of adsorbents	Cosmetics with specific active ingredients	[151]
Dispersive solid phase extraction	Dispersive solid-phase extraction (dSPE)	Simple, low cost, and quick processing time	May require more solvent	Solid and semi-solid cosmetics, like foundations and eyeshadows	[5]
	Matrix solid-phase dispersion (MSPD)	Integrates sample dispersion and extraction, improving efficiency	Technically more complex	Solid and semi-solid samples	[156]
	Dispersive liquid–liquid microextraction (DLLME)	Rapid, low cost, and requires minimal solvent	High specificity in solvent selection	Extraction of small organic compounds	[13]
Ultrasound-assisted extraction	UA-MSPD	Ultrasound improves extraction efficiency	Potential damage to sensitive components	A broad range of cosmetic products	[134]
	Ultrasound-assisted extraction (UAE)	Ultrasound speeds up the extraction process, saving time	Requires specific equipment	A broad range of cosmetic products	[129]
Liquid–liquid extraction	Liquid–liquid extraction (LLE)	Traditional method with wide applicability	High solvent consumption, potentially less environmentally friendly	A broad range of cosmetic products	[138]
	Liquid phase microextraction (LPME)	Low solvent usage, more environmentally friendly	May require specialized equipment	Extracts easily separable from water with organic solvents	[153]
Cloud point extraction	Cloud point extraction (CPE)	Eco-friendly, does not require organic solvents	May need temperature control	Samples containing surfactants.	[142]
	Co-precipitation-assisted CPE	Enhances extraction efficiency, reduces solvent usage	Potentially more complex operation steps	Samples requiring simultaneous removal of various impurities	[143]
Stir-bar adsorption extraction	Stir-bar sorptive extraction (SBSE)	Reusable, minimal solvent required	Potential for desorption of analytes during processing	Volatile and semi-volatile organic compounds analysis in perfumes and essential oils	[139]
Pressurized liquid extraction	Pressurized liquid extraction (PLE)	Increased extraction efficiency and speed due to the use of high pressure	High equipment cost	Solid and semi-solid samples	[157]
	Accelerated solvent ex-traction (ASE)	Fast, saves solvents and time	Requires specialized equipment	Solid or viscous samples	[158]
Other extraction methods	Digestion	Complete breakdown of samples, suitable for mineral analysis	Can destroy some organic components	Samples requiring complete decomposition, like for mineral content analysis	[152]
	Ashing	Removes organic matter, useful for inorganic component analysis	Not suitable for organic component analysis	Analysis of inorganic components like heavy metals	[161]

## 4. Conclusions

The rapid development of the cosmetics industry has not only unleashed significant market potential but also given rise to a series of challenges. The proliferation of illegal additives, regulatory delays, and the profit-driven practices of unscrupulous traders have led to the circulation of some cosmetics containing these harmful substances, posing a threat to consumer health. Despite advancements in analytical detection technologies in the food and pharmaceutical sectors, their application in cosmetics is hampered by the complexity of cosmetic formulations.

In this article, we categorize cosmetics into four main types: emulsified, liquid, powdered, and wax-based cosmetics, and provide a detailed analysis of their primary constituents, characteristics, and additive thresholds. A thorough understanding of cosmetic composition is crucial for selecting and optimizing pretreatment methods. For example, emulsified cosmetics, rich in emulsifiers and thickeners, are amenable to techniques such as UA–MSPD and dSPE. Special attention to solubility issues is necessary when employing SPE techniques. In contrast, liquid cosmetics, with their less complex matrices, are well-suited to techniques such as LLE, CPE, SPE, and SBSE. Similarly, powdered cosmetics, replete with insoluble minerals, are efficiently processed using techniques such as MSPD, PLE, and ASE, thereby avoiding the need for dissolution. Lastly, for wax-based cosmetics, alternatives to solvent use, such as mechanical stirring or ashing, are preferable for safety and toxicity concerns.

Furthermore, we provide an overview of the illegal additives in cosmetics, including their types, functions, and potential presence in various cosmetic products. Mastery of this information is vital for selecting appropriate analytical detection methods. Therefore, we also introduce commonly used analytical techniques, such as UV/VIS, IR, MS, NMR, GC, HPLC, AAS, and AFS. In-depth knowledge of cosmetic constituents and illegal additives is essential for cosmetic analysis.

Finally, in the third section, we comprehensively review scientific research in the field of cosmetic analysis and detection and categorize findings according to cosmetic types. This categorization aids in a clearer understanding of the objectives and focus of this review.

## 5. Future Perspectives

Future research should focus on devising more appropriate and eco-friendly extraction methods that align with specific matrix compositions and advancing compact and portable extraction devices to minimize sample and solvent usage while reducing time spent. The integration of nanotechnology has offered promising materials, such as metal–organic frameworks (MOFs) [162], molecularly imprinted polymers (MIPs) [163], and magnetic nano-sorbents (MNSs) [164], whose vast surface areas and selective binding can be leveraged for enhanced detection. Additionally, rapid and on-site analytical strategies, such as surface-enhanced Raman spectroscopy (SERS) [165] and electrochemical techniques [166], have been employed in the food and environmental sectors. The detection of illicit additives in cosmetics is analogous to compounds analysis in these areas. For instance, wastewater and liquid cosmetics, both characterized by higher fluidity, exhibit similar properties; likewise, the study of more viscous substances, like edible oils, shares methodological parallels with the analysis of emulsified cosmetic. Additionally, the analysis of solid materials, such as traditional Chinese medicinal herbs, can offer novel insights and techniques for the pretreatment of solid cosmetics. Therefore, these technologies guide the development of detection techniques for unauthorized additives in cosmetics.

## Figures and Tables

**Table 1:** Thresholds for cosmetic ingredient additions.

Ingredients	Thresholds	Ingredients	Thresholds	Ingredients	Thresholds	Ingredients	Thresholds	Ingredients	Thresholds
Preservatives		Ubidecarenone (**24**)	N.D. ^[f]^	Cetylicalcohol (**45**)	N.D.	Linoleic acid diethanolamide (**68**)	10% in rinse-off products [108]	PMMA	N.D. ^[f]^
Methylparaben (**1**)	0.4% (as acid) for single ^[a]^; 0.8% (as acid) for mixtures _[a]_	pH Adjusters				Polyethylene glycol monocetyl ether (**69**)	0.4–6.7% ^[d]^; 0.345–6.25% ^[e]^		
Propylparaben (**2**)	0.14% (as acid) for single ^[a]^ 0.8% (as acid) for mixtures ^[a]^	Triethanolamine (**25**)	8% ^[c]^	Tween 60 (**46**)	25% ^[e]^	Cetyl palmitate (**70**)	12.5% ^[e]^	Sorbitan esters	3–20% ^[d]^; 1–20.7% ^[e]^
Butylparaben (**3**)		Citric acid (**26**)	0.2% ^[a]^	Span 60 (**47**)	5% [109]	Myristyl dimethylamine oxide (**71**)	0.39% ^[d]^	Propylene glycol	89.493% ^[d]^; 66.19% ^[e]^
Methylisothiazolinone (**4**)	0.0015% ^[a]^	Sodium citrate (**27**)	N.D. ^[f]^	Stearyl alcohol polyether-2 (**48**)	8.95% ^[d]^; 8% ^[e]^	Hexadecylbetaine (**72**)	10% ^[d]^	PEG-8	69% ^[d]^; 60% ^[e]^
Chloromethylisothiazolinone (**5**)		Succinic acid (**28**)	10% ^[d]^; 1% ^[e]^	Polyethylene glycol octadecyl ether (**49**)	1.7% ^[d][e]^	Potassium oleate (**73**)	30.4% ^[d]^	Fillers	
Imidazolidinyl urea (**6**)	0.6% ^[a]^	Disodium succinate (**29**)	1.6% ^[e]^	Glycerine monostearate (**50**)	No Limits [110]	Cellulose (**74**)	18% ^[e]^	Mica	No limits [111]
DMDMH (**7**)	0.6% ^[a]^	Potassium phosphate (**30**)	0.88% ^[d]^	Polyglycerol-10 laurate (**51**)	3.5% ^[d]^; 3% ^[e]^	Alginic acid (**75**)	0.33915% ^[e]^	Kaolin	83.333% ^[d]^; 67.742% ^[e]^
Chlorphenesin (**8**)	0.3% ^[a]^	Chelators		Eumulgin SG (**52**)	67.12% ^[d]^; 2% ^[e]^	Hectorite (**76**)	5% ^[d]^; 3.8% ^[e]^	Calcium carbonate	No limits [112]
2-phenoxyethanol (**9**)	1.0% ^[a]^	Disodium EDTA (**31**)	5% ^[e]^	Montanov™ 82 (**53**)	3% [113]	Solvents		Talc	No limits [114]
1,2-pentanediol (**10**)	21.29 ^[e]^	Citric acid (**32**)	0.2% ^[a]^	Thickeners		Water	No limits [115]	Silica	100% ^[e]^
Sorbic acid (**11**)	0.6% (as acid) ^[a]^	Phosphoric acid (**33**)	2.55% ^[d]^; 0.6 ^[e]^	Glycerin (**54**)	98.525% ^[d]^; 62.1% ^[e]^	Ethanol	5% ^[a]^	Pearl	49.51% ^[d]^; 0.1% ^[e]^
Potassium sorbate (**12**)	0.6% (as acid) ^[a]^	Fragrances		1,2-butanediol (**55**)	8% ^[d]^; 6% ^[e]^	Ethyl acetate	N.D.	Nylon	25% ^[d]^; 3–59.5 ^[e]^
Sodium benzoate (**13**)	2.5% (as acid) for rinse-off products; 1.7% for oral care products ^[a]^	Linalool (**34**)	1.25% ^[d]^; 1% ^[e]^	1,3-butanediol (**56**)	87.98% ^[e]^	Toluene	33% [116]	Colorants	
Levulinic acid (**14**)	5% ^[e]^	Vanillin (**35**)	1.2% ^[e]^	Sodium *L*-pyroglutamate (**57**)	20% ^[e]^	Propellants		Iron oxides	No limits [117]
Anisic acid (**15**)	0.96% ^[e]^	Cinnamaldehyde (**36**)	0.016% ^[d]^	Panthenol (**58**)	40% ^[e]^	Propane	58.7% ^[d]^; 36.544% ^[e]^	Zinc oxide	No limits in non-nano particle form [118]
Antioxidants		Eugenol (**37**)	0.036% ^[d]^; 0.031 ^[e]^	Sorbitol (**59**)	51.46% ^[d]^; 38.849% ^[e]^.	Butane	70.045% ^[d]^; 56% ^[e]^	Titanium dioxide	25% ^[a]^
Butylated hydroxytoluene (**16)**	0.001% for mouthwash; 0.01% for toothpaste; ^[b]^	Oil-based Constituents		Betaine (**60**)	20% ^[e]^	Isobutane	81.522% ^[e]^	Mica	No limits [119]
Butylated hydroxyanisole (**17**)	0.1% [120]	Grape seed oil (**38**)	No Limits [121]	Sodium lactate (**61**)	10% [122]	Carbon dioxide	50% ^[e]^	Waxes	
Tertiary-butylhydroquinone (**18**)	0.1% [123]	Petrolatum (**39**)	75.175% ^[d]^	Sodium hyaluronate (**62**)	74.993% ^[d]^; 1% ^[e]^	Nitrogen	40.476% ^[e]^	Carnauba	5% ^[e]^
Propyl gallate (**19**)	8.0854% ^[d]^; 1.5% ^[e]^	Isopropyl palmitate (**40**)	79.69% ^[e]^	Ceramides (**63**)	22.5% ^[e]^	Binders		Candelilla	30% ^[e]^
Vitamin C (**20**)	N.D. ^[f]^	Isopropyl myristate (**41**)	78.278% ^[d]^; 42% ^[e]^	Lauryl alcohols (**64**)	15% ^[d]^; 3.5% ^[e]^	Beeswax	50% ^[e]^	Jojoba	5% ^[e]^
Hyaluronic acid (**21**)	2% [124]	Isononyl isononyl ester (**42**)	71.4% ^[e]^	Myristyl alcohols (**65**)	12% ^[d]^; 7.02% ^[e]^	Keratin	1% ^[d]^	Bees	50% ^[e]^
Vitamin E (**22**)	33.702% ^[e]^	Lecithin (**43**)	20.008% ^[d]^; 14% ^[e]^	Linolenic acid (**66**)	13.3% ^[e]^	Liquid paraffin	99.788% ^[e]^	Japan waxes	8% ^[e]^
Squalene (**23**)	82% ^[c]^; 2% ^[e]^	Squalane (**44**)	48.98% ^[e]^	Coconut monoethanolamide (**67**)	1.24% ^[d]^	Silicone oils	N.D. ^[f]^		

a. European Union regulations (EC) No 1223/2009; b. Commission Regulation (EU) 2022/2195; c. Safety and Technical Standards for Cosmetics of China (2015 Edition); d. Highest historical usage of drenching products (rinse-off products) from list of substances allowed in cosmetic products of China (2021 Edition); e. Highest historical usage of residency category products (leave-on products) from list of substances allowed in cosmetic products of China (2021 Edition); f. N.D. indicates no data.

## Data Availability

Not applicable.
